# Time-resolved myocardial perfusion MRI with reduced data acquisition window, improved spatial coverage, resolution and SNR

**DOI:** 10.1186/1532-429X-11-S1-P252

**Published:** 2009-01-28

**Authors:** Lan Ge, Aya Kino, Mark Griswold, Charles Mistretta, Daniel Lee, James Carr, Debiao Li

**Affiliations:** 1 Northwestern University, Evanston, IL USA; 2 Case Western Reserve University, Cleveland, OH USA; 3 University of Wisconsin-Madison, Madison, WI USA; 4 Northwestern Memorial Hospital, Chicago, IL USA

**Keywords:** Cardiac Cycle, Composite Image, Reduce Motion Artifact, Slide Window Method, Cardiac Motion Artifact

## Introduction

Perfusion MRI is a promising technique to detect ischemic heart disease. Single-shot imaging is currently used to acquire 3 slices with a temporal resolution of one time frame per cardiac cycle. However, this technique is challenged by the cardiac motion artifacts, limited spatial coverage, resolution and SNR. Reduced imaging time per slice will allow greater coverage of the heart and reduced motion artifacts, especially during stress imaging with high heart rates. Parallel imaging methods have been applied in myocardial perfusion MRI, but the acceleration factors are limited to 2–3 because of SNR considerations. Time-resolved data acquisition with shortened data acquisition window can be performed with Conjugate-gradient Highly Constrained Backprojection reconstruction (CG-HYPR) [1, 2]. A combination of sliding composite images and CG-HYPR method allows vast undersampling, increased spatial coverage, resolution and SNR while preserving the temporal resolution of one frame per heartbeat and dynamic blood and myocardial signal intensity changes. In this work, we compared this new method with the conventional clinical protocol for myocardial perfusion in healthy volunteers.

## Methods

An ECG-triggered, 2D multi-slice FLASH sequence with radial *k*-space sampling was used in this study. The *k*-space was acquired in a segmented interleaved fashion. The "composite images" were reconstructed by a sliding window method with full *k*-space data. CG-HYPR method was used to reconstruct time-resolved images (one image per cardiac cycle) combining signal intensity information from the undersampled radial projections, and the structural information from the sliding composite images whose center corresponds to the current cardiac cycle.

Six healthy volunteers were scanned during a single breath-hold in a 3 T system. The segmented SR-prepared multi-slice radial FLASH sequence was performed with the following parameters: TR/TE/flip-angle = 3.2/1.6 ms/10°, matrix = 160 × 192, saturation pre-pulse delay = 50 ms, number of slices = 6, and spatial resolution = 1.4 × 1.4 × 8 mm. Six datasets were collected over 60 heartbeats, with the data from 20–30 heartbeats is usable during breath-hold. Each of the sliding composite images were reconstructed over 10 cardiac cycles with 16 projections per cardiac cycle and 51 sliding composite images. Time-resolved images with the resolution of one image per cardiac cycle were generated after the CG-HYPR processing.

To verify the signal changes, a conventional scan was performed with the same contrast injection scheme. The imaging parameters were: TR/TE/flip-angle = 2.2/1.1/10°, matrix = 106 × 192 (collected lines/heartbeat = 63), GRAPPA factor = 2, saturation pre-pulse delay = 40 ms, number of slices = 3, and spatial resolution = 3.1 × 2.3 × 8. The dynamic signal changes and the SNR of images were compared between the sliding CG HYPR images and the conventional images.

## Results

**Figure 1:**
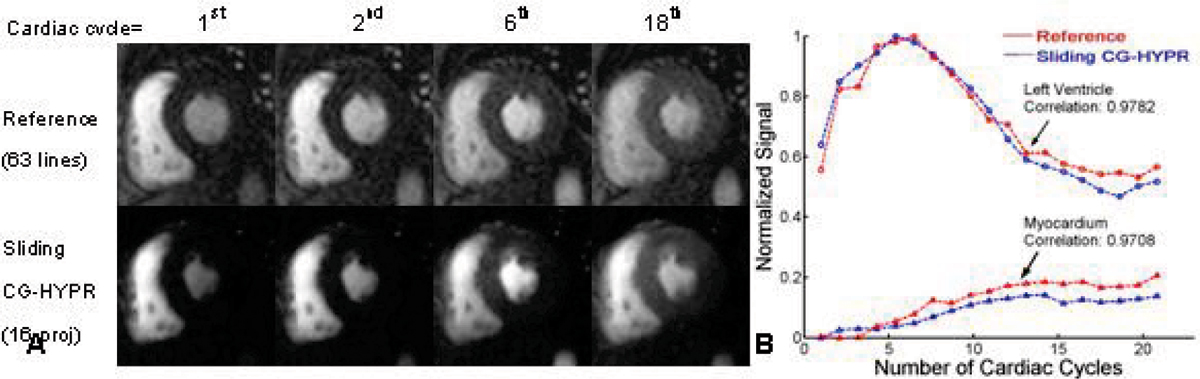
**One typical example of the 6 volunteer studies**. A) Comparison of the images from conventional method and sliding CG-HYPR. Blood signal change is observed in sliding CG-HYPR perfusion images, and comparable to the traditional images. B) Comparison of the left ventricle and myocardium signal changes vs. time.

**Figure 2:**
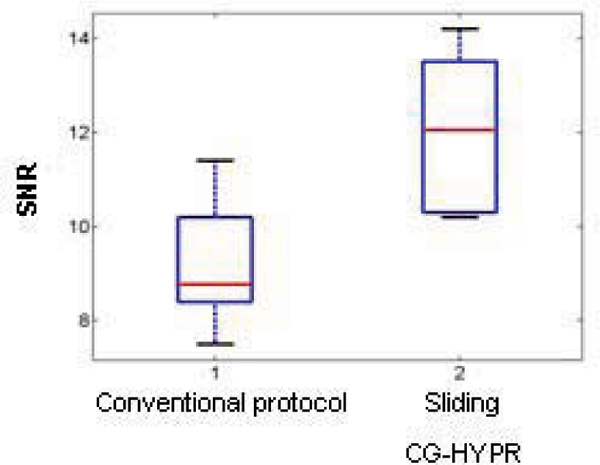
**SNR comparison between images from sliding CG-HYPR and conventional protocol**.

Although with only 16 projections per slice per cardiac cycle, the signal change distribution of the sliding CG-HYPR images are similar to the reference images by conventional protocol (Figure 1A), which can be further shown by the quantitative measurements (Figure 1B). The mean correlation coefficients of the 6 studies for signal changes between the two methods were 0.9672 and 0.9423 for blood and myocardial signals, respectively. The averaged SNR images around blood signal peak from the two methods were calculated and compared. The CG-HYPR images had significantly higher SNR than conventional protocol (p < 0.05, t-test) (Figure 2). The spatial coverage and spatial resolution were improved roughly by a factor of 2.

## Conclusion

This work demonstrated the feasibility of SCG-HYPR for accelerated myocardial perfusion imaging. The acquisition time can be reduced dramatically, allowing an increased number of slices and reduced motion artifacts. Spatial resolution and SNR can be improved while preserving the signal change accuracy.
